# Influence of clinicopathological characteristics and comprehensive treatment models on the prognosis of small cell carcinoma of the cervix: A systematic review and meta-analysis

**DOI:** 10.1371/journal.pone.0192784

**Published:** 2018-04-11

**Authors:** Qian Zhang, Yao Xiong, Jiaxiang Ye, Liying Zhang, Li Li

**Affiliations:** 1 Department of Gynecologic Oncology, The Affiliated Tumor Hospital of Guangxi Medical University, Nanning, China; 2 Department of Medical Oncology, The Affiliated Tumor Hospital of Guangxi Medical University, Nanning, China; Duke Cancer Institute, UNITED STATES

## Abstract

Small cell carcinoma of the cervix (SCCC) is a rare primary neuroendocrine cervical carcinoma with a high degree of invasiveness. SCCC is prone to early-stage lymph node and distant metastases and characterized by a poor prognosis. Currently, there is no standard treatment. This study aimed to evaluate the clinicopathological factors and treatment models that influence SCCC prognosis through a systematic review and meta-analysis, to improve the diagnosis and treatment of SCCC. A comprehensive search was performed in multiple medical literature databases to retrieve studies on the clinical prognosis of SCCC published in China and abroad as of March 1, 2017. Twenty cohort studies with 1904 patients were analyzed. Meta-analysis showed statistical significance for the following factors: FIGO staging (hazard ratio [HR] = 2.63, 95% confidence interval [CI]: 2.13–3.24; odds ratio [OR] = 3.72, 95% CI: 2.46–5.62), tumor size (HR = 1.64, 95% CI: 1.25–2.15), parametrial involvement (HR = 2.40, 95% CI: 1.43–4.05), resection margin (HR = 4.09, 95% CI: 2.27–7.39), lymph node metastasis (OR = 2.09, 95% CI: 1.18–3.71), depth of stromal invasion (HR = 1.99, 95% CI: 1.33–2.97), neoadjuvant chemotherapy (HR = 2.06, 95% CI: 1.14–3.73), and adjuvant chemotherapy (HR = 1.63, 95% CI: 1.26–2.12; OR = 1.48, 95% CI: 1.02–2.16). FIGO staging, tumor size, parametrial involvement, resection margin, depth of stromal invasion, and lymph node metastasis can be used as clinicopathological characteristics for the prediction of SCCC prognosis. Neoadjuvant chemotherapy tended to improve prognosis. Our findings suggest that neoadjuvant chemotherapy plus adjuvant chemotherapy may be the preferred strategy. However, adjuvant radiotherapy appeared to cause no significant improvement in prognosis. Therefore, the clinical application of radiotherapy and the relationship between radiotherapy and clinicopathological factors need to be re-examined. The results of this study should be validated and developed in formal, well-designed multicenter clinical trials.

## Introduction

Small cell carcinoma of the cervix (SCCC) is a rare neuroendocrine cervical carcinoma that accounts for less than 3% of all cervical cancers[1]. These tumors are characterized by a high incidence of early-stage lymph node and distant metastases and poorer prognoses than other cervical cancers. In previous studies, lymphovascular space invasion and pelvic lymph node metastasis were found at the time of diagnosis in 60.0–82.0% of SCCC cases[2]. In addition, this rare disease tends to rapidly metastasize to lateral and distant areas, such as the lungs, liver, brain, bone, and lymph nodes, reducing the overall survival (OS) of patients and leading to treatment failure in most cases[3].

Small cell carcinoma of the cervix is a highly invasive neuroendocrine tumor. Its clinical manifestations and presentations are similar to those of other cervical cancers. The most common clinical manifestations of SCCC are irregular bleeding or contact bleeding in the vagina, with or without abnormal vaginal discharge, and neoplasms are detected in the cervix through specialized examination. Previous retrospective analyses suggested significant differences between SCCC and common squamous cell carcinoma or adenocarcinoma of the cervix in terms of histology, pathology, and biological behavior. Primary small cell cervical carcinoma may not infiltrate the surface of the cervix, but instead may directly infiltrate the cervical stroma in a diffuse manner. Therefore, the associated rates of lymphatic vessel invasion and lymph node metastasis are significantly higher than in other tumors of the cervix, leading to high rates of early recurrence and poor prognoses[4]. Lee et al. [5] conducted a 1:2 matched, case-control study in 32 patients with SCCC and 64 patients with squamous cell carcinoma of the cervix, and found that the recurrence rate of SCCC was 59.4%, with the lung, bone, and liver being the common sites of distant metastasis, and the progression-free and OS were significantly shorter in SCCC patients than in those with squamous cell carcinoma of the cervix.

Given the poor prognosis of SCCC, determining prognostic factors for survival is paramount to improving treatment strategies. However, due to the scarcity of patients and long recruitment times, most SCCC studies are only composed of small case series and reports, which makes it exceedingly difficult to conduct randomized controlled clinical trials to determine the optimal therapeutic strategy. The aim of this study was to determine the influence of risk factors and treatment models on the prognosis of SCCC by conducting a meta-analysis on published literature retrieved by a comprehensive database search.

## Materials and methods

### Literature retrieval

A comprehensive search was performed in the PubMed database, Excerpta medical database (Embase), Cochrane Library, Wanfang standards database (WFSD), China national knowledge infrastructure (CNKI) database, and China biology medicine (CBM) database to retrieve literature related to SCCC published before March 1, 2017. The retrieval strategies were as follows:

1.1 “Small cell carcinoma of the cervix” OR “Small cell neuroendocrine carcinoma of the cervix” [Mesh]
1.2 “Clinical” [Mesh] AND “Factor”
1.3 “Clinicopathological” [Mesh] AND “Characteristics”
1.4 “Treatment” [Mesh]
1.5 “Prognosis” [Mesh]
1.6 Strategies 1 through 5
1.7 “Chemotherapy” or “Radiotherapy” or “Neoadjuvant chemotherapy”
1.8 Strategies 1 and 7
1.9 Strategy 1 and “Combination therapy”

### Inclusion and exclusion criteria

The inclusion criteria were as follows: controlled clinical studies and cohort studies, written in English or Chinese; the study subjects of the reports were patients with SCCC confirmed by pathological diagnosis without age or racial restrictions; all reports studied the influence of clinicopathological characteristics and treatment models on the prognosis of SCCC; reports provided hazard ratios (HRs) or odds ratios (ORs) related to the prognosis of SCCC, and, as SCCC cases are relatively rare, ORs provided in the literature were considered approximations of HRs; if a HR and its 95% confidence interval (CI) were provided, they could be combined as appropriate; if the literature did not provide the HR but provided the survival rate or Kaplan-Meier curve, they could be used to estimate the HR, 95% CI, and the number of patients achieving long-term survival; and the original report was related to the clinical prognosis of SCCC and published as of March 1, 2017.

**Fig 1:**
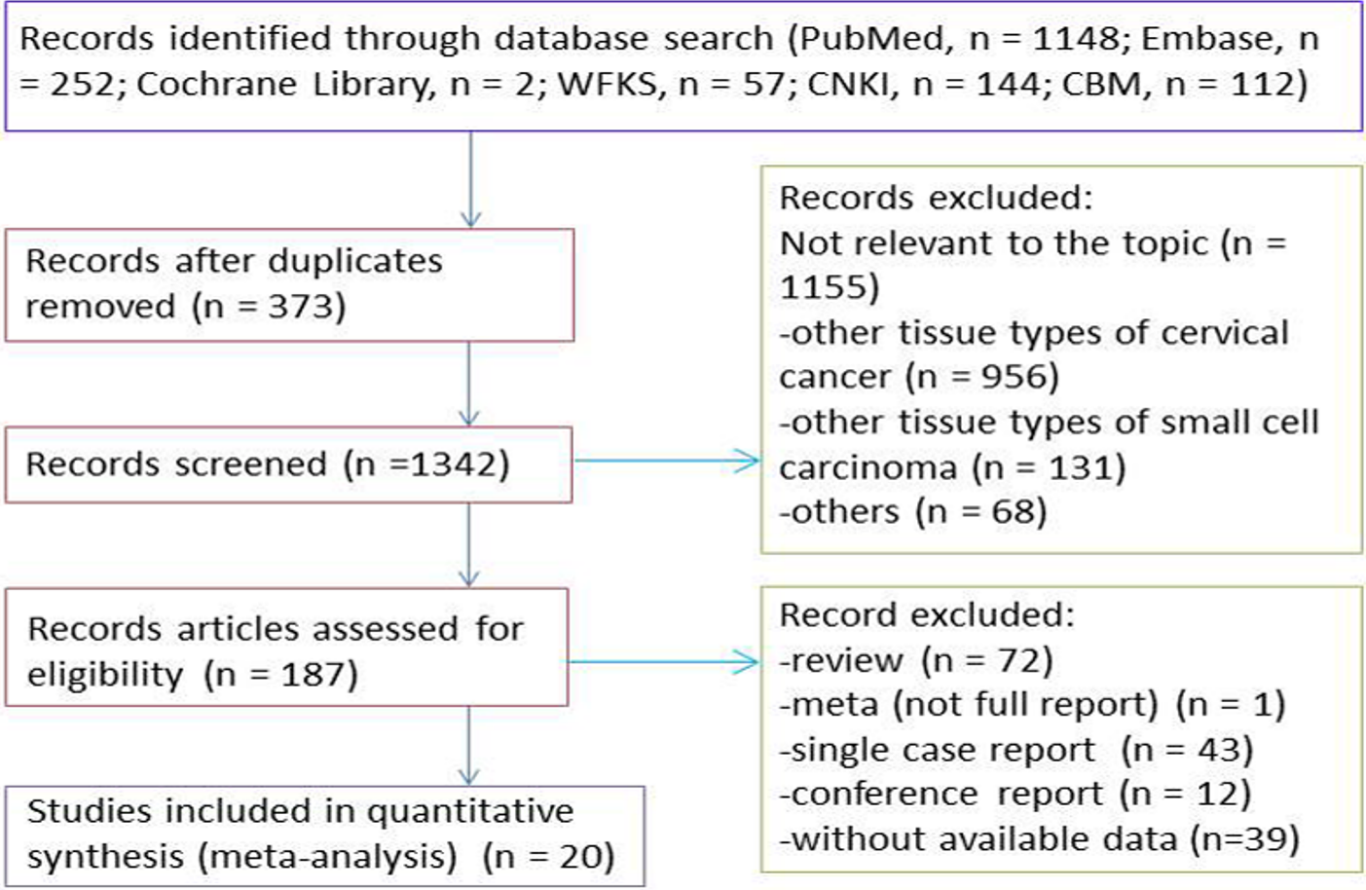
Studies eligible for inclusion in the meta-analysis. CKNI: China national knowledge infrastructure; CBM: China biology medicine.

The exclusion criteria were as follows: case reports and comments; other histological types of cervical cancers not classified as SCCC; literature on basic research and animal studies; and literature that did not study factors related to the prognosis of SCCC or did not provide sufficient information for the calculation of the HR, 95% CI, or number of cases achieving long-term survival. The literature screening process is illustrated in Fig 1.

### Data extraction

The following data were carefully extracted and quality-checked: name of the first authors, year of publication, country of origin, number of patients, median age, median survival time, median follow-up time, and clinical outcomes. The basic characteristics of the studies are shown in Table 1.

**Table 1:** Studies included in the meta-analysis.

Study (au,y,ref)	Country	Age (y)	Survival time (m)	Follow-up time (m)	N	Outcome
Chan 2003[6]	America	42 (28–79)	NR	NR	34	OS
Chen 2008[7]	America	45	NR	14.5(29.5-?)	290	OS
Lee 2008[8]	Korea	45.8 (32–87)	54 (6–133)	44	68	OS
Li 2008[9]	China	42.5 (24–65)	22 (1–110)	(10-?)	18	Survival rate
Huang 2009[10]	Taiwan	45 (26–84)	NR	25 (4–143)	18	DFS/OS
Yin 2009[11]	China	41.3 (25–83)	45.0	(29–115)	20	Kaplan-Meier
Lee 2010[5]	Korea	45 (27–70)	30.6	NR	32	OS
Long 2012[12]	China	46.0 (32–68)	NR	NR	20	Survival rate
Cohen 2012[13]	America	45 (20–87)	NR	NR	188	OS
Intaraphet.S2014[14]	Thailand	44.3(33.4–55.2)	47.8(24.7–200.1)	NR	130	OS
Wang 2012[15]	Taiwan	42 (25–89)	24.8	51.2(1.3–228.7)	179	CSS/OS
Kuji 2013[16]	Japan	40 (20–84)	NR	57 (4–126)	52	Kaplan-Meier
Liao 2012[17]	China	40 (18–83)	23	NR	293	OS
Lee 2014[18]	Korea	50 (27–84)	40.7(5.0–218.7)	NR	102	TTP/OS
Lei 2015[1]	China	40.4(33.4–47.4)	55	NR	38	DFS/OS
Zhou 2015[19]	China	37 (23–85)	NR	30.5 (4–250)	118	CSS/OS
Liu 2015[20]	China	45 (30–60)	27(4–95)	NR	21	Kaplan-Meier
Xia 2016[21]	China	39 (22–51)	NR	(2–65)	27	Survival rate
Zhou 2016[22]	China	40.5 (22–90)	NR	31 (5–237)	208	CSS/OS
Xie 2017[23]	China	41 (25–67)	30.7	20.6	48	OS

NR, not reported; OS, overall survival; DFS, disease-free survival; CSS, cancer-specific survival; TTP, time-to-progression.

### Quality assessment

The Newcastle-Ottawa Scale (NOS) was used for all qualitative studies. The NOS provides a standard quality assessment for cohort studies based on 3 aspects, namely, assessment of the selection of exposed and unexposed cohorts, comparability between the 2 cohorts, and assessment of the results[24]. A study can be awarded a maximum of one star (★) for each numbered item within the Selection and Outcome categories. A maximum of two stars (★ ★) can be given for Comparability. If all criteria are met, nine stars are rewarded. These results are shown in Table 2.

**Table 2:** Results of quality assessment using the Newcastle-Ottawa Scale for cohort studies.

Study (au,y)	A1	A2	A3	A4	B	C1	C2	C3	Score
Chan 2003	★	★	★	★	★	★	★	★	8
Chen 2008	★	★	★	★	★ ★	★	★	★	9
Lee 2008	★	★	★	★	★	★	★	★	8
Li 2008	★	★	★	★	★	★	★	一	7
Huang 2009	★	★	★	★	★	★	★	★	8
Yin 2009	★	★	★	★	★	★	★	一	7
Lee 2010	★	★	★	★	★ ★	★	★	一	8
Long 2012	★	★	一	★	★	★	★	一	6
Cohen 2012	★	★	★	★	★ ★	★	★	一	8
Intaraphet.S 2014	★	★	★	★	★	★	★	★	8
Wang 2012	★	★	★	★	★	★	★	一	7
Kuji 2013	★	★	一	★	★	★	★	★	7
Liao 2012	★	★	★	★	★	★	★	★	8
Lee 2014	★	★	★	★	★	★	★	一	7
Lei 2015	★	★	★	★	★	★	★	★	8
Zhou 2015	★	★	一	★	★ ★	★	★	★	8
Liu 2015	★	★	★	★	★	★	★	一	7
Xia 2016	★	★	一	★	★	★	★	一	6
Zhou 2016	★	★	★	★	★ ★	★	★	★	9
Xie 2017	★	★	★	★	★ ★	★	★	★	9

A1, Representation of the exposed cohort; A2, Selection of the non-exposed cohort; A3, Ascertainment of exposure to implants; A4, Demonstration that the outcome of interest was not present at the start of the study; B, Comparability of cohorts on the basis of the design or analysis; C1, Assessment of outcome; C2, Was follow-up long enough for outcomes to occur; C3, Adequacy of follow-up of outcome; A, B, C represent Selection, Comparability, Outcome, respectively; ★ and ★ ★indicate compliance with the requirements of the definition, for which specific meaning see **S1 Text**.

### Data analysis

For the prognosis of SCCC, analysis was performed on overall survival (OS), with a combined analysis performed on the HR and 95% CI provided in each article. A heterogeneity analysis was performed using the Q-test and I^2^ values. If the heterogeneity test showed P > 0.1 and I^2^ < 50%, the study heterogeneity was deemed relatively small, and a fixed effects model was adopted. If P < 0.1 and I^2^ > 50%, it was deemed that the heterogeneity was relatively large, and the random effects model was adopted to perform the combined analysis. If heterogeneity was still present, subgroup analysis or sensitivity analysis was used to explore the source of heterogeneity in the results. Publication bias was assessed by visual inspection of the asymmetry of the funnel plot, as well as by Begg’s and Egger’s tests using Stata 12.0 software (StataCorp LLC., College Station, Texas, USA). The trim and fill method was used to correct publication bias, if any existed.

## Results

**Fig 2:**
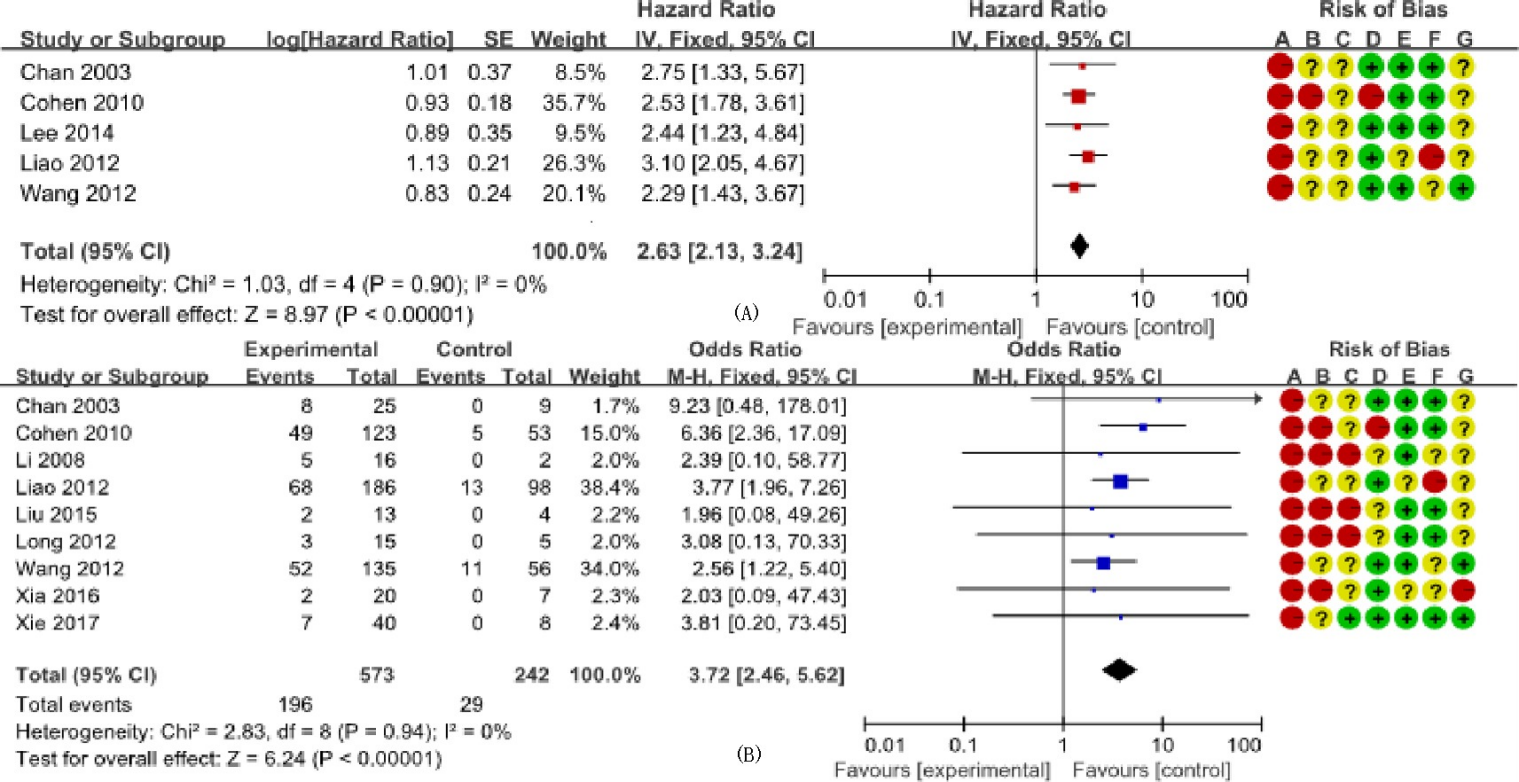
FIGO (International Federation of Gynecology and Obstetrics) staging. **Forest plot of FIGO staging (Ia-IIa/IIb-IV) and overall survival of small cell carcinoma of the cervix patients.** (A) The hazard ratios of the analyzed studies; and (B) the odds ratios of the analyzed studies. SE: standard error; CI: confidence interval.

**Fig 3:**
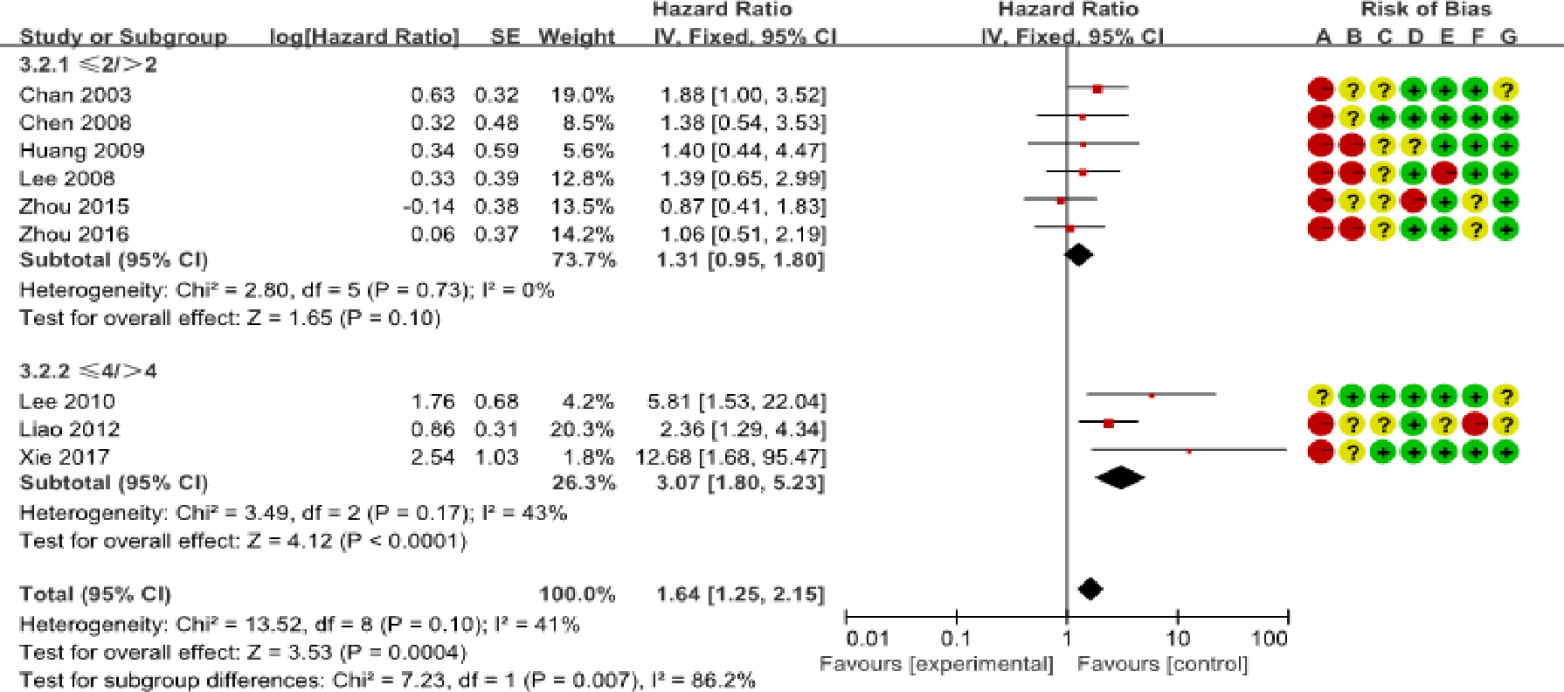
Tumor size. **Forest plot of tumor size and overall survival of small cell carcinoma of the cervix patients.** SE: standard error; CI: confidence interval.

**Fig 4:**
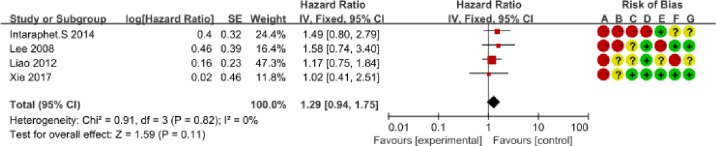
Forest plot of tumor homology and overall survival of small cell carcinoma of the cervix patients. SE: standard error; CI: confidence interval.

**Fig 5:**
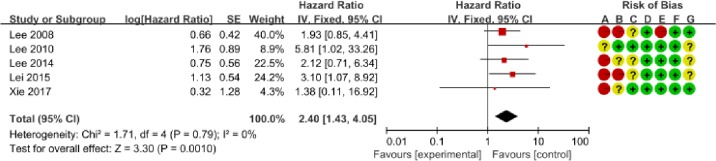
Forest plot of parametrial involvement (-/+) and overall survival of small cell carcinoma of the cervix patients. SE: standard error; CI: confidence interval.

**Fig 6:**
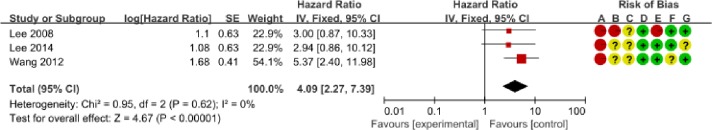
Forest plot of resection margin (-/+) and overall survival of small cell carcinoma of the cervix patients. SE: standard error; CI: confidence interval.

**Fig 7:**
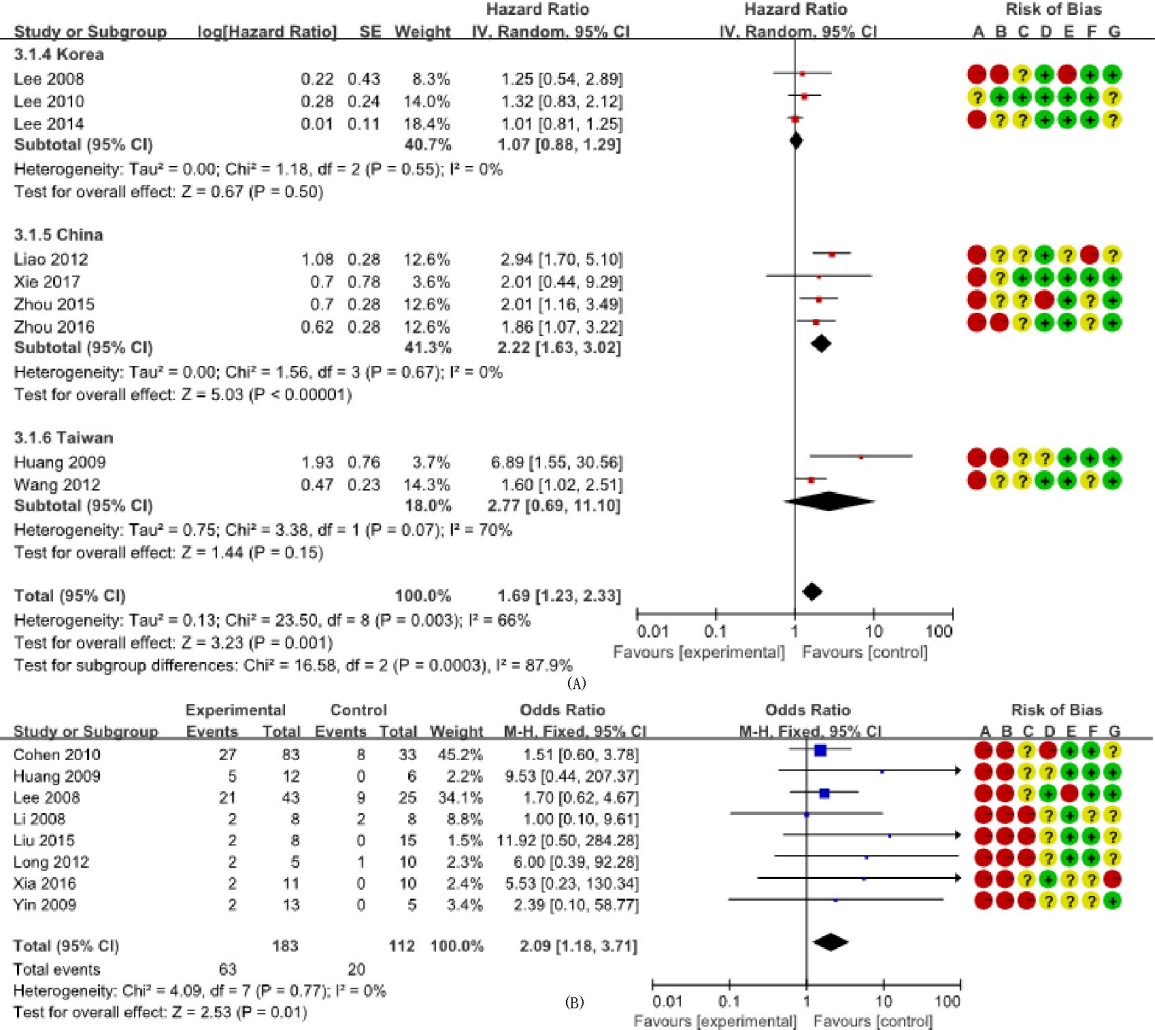
Forest plot of lymph node metastasis and either overall survival and survival rate of small cell carcinoma of the cervix patients. (A) studies reporting hazard ratios; and (B) studies reporting odds ratios. SE: standard error; CI: confidence interval.

**Fig 8:**
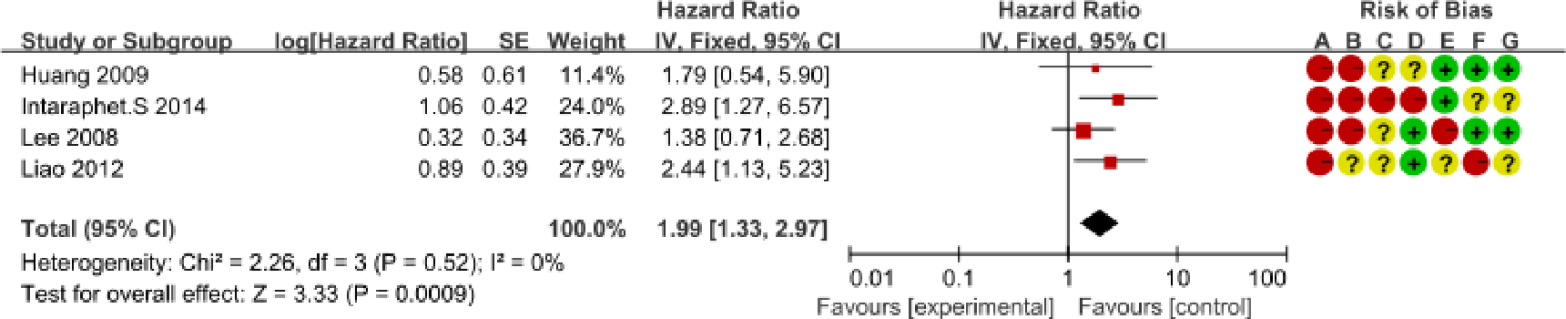
Forest plot of depth of stromal invasion (>2/3/≤1/3/) and overall survival of small cell carcinoma of the cervix patients. SE: standard error; CI: confidence interval.

**Fig 9:**
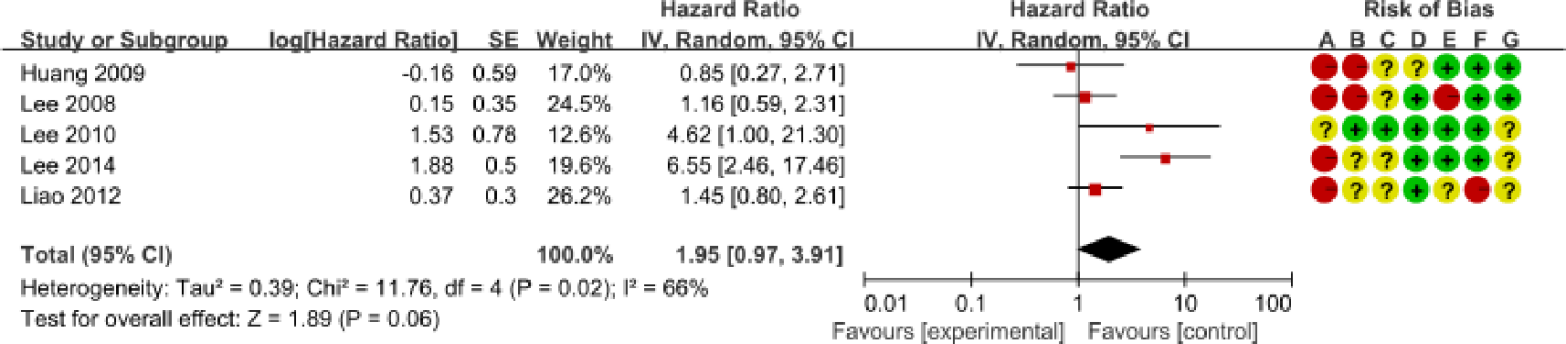
Forest plot of lymphovascular space invasion (-/+) and overall survival of small cell carcinoma of the cervix patients. SE: standard error; CI: confidence interval.

**Fig 10:**
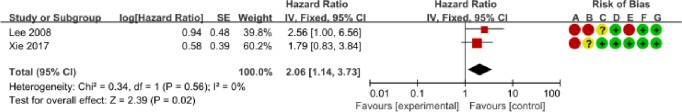
Forest plot of neoadjuvant chemotherapy (+/-) and overall survival of small cell carcinoma of the cervix patients. SE: standard error; CI: confidence interval.

**Fig 11:**
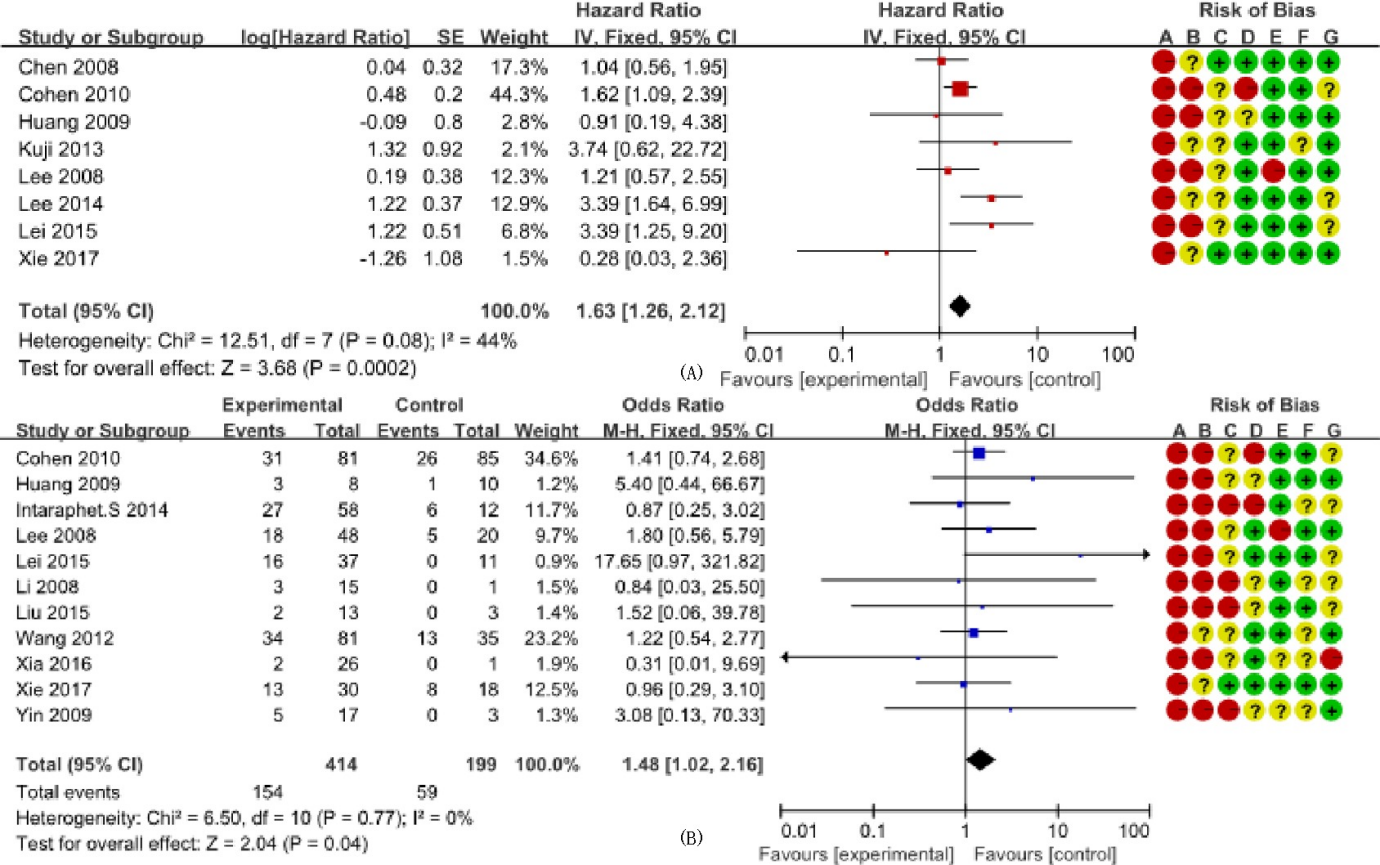
Forest plot of adjuvant chemotherapy (+/-) and overall survival and survival rate of small cell carcinoma of the cervix patients. (A) studies reporting hazard ratios; and (B) studies reporting odds ratios. SE: standard error; CI: confidence interval.

**Fig 12:**
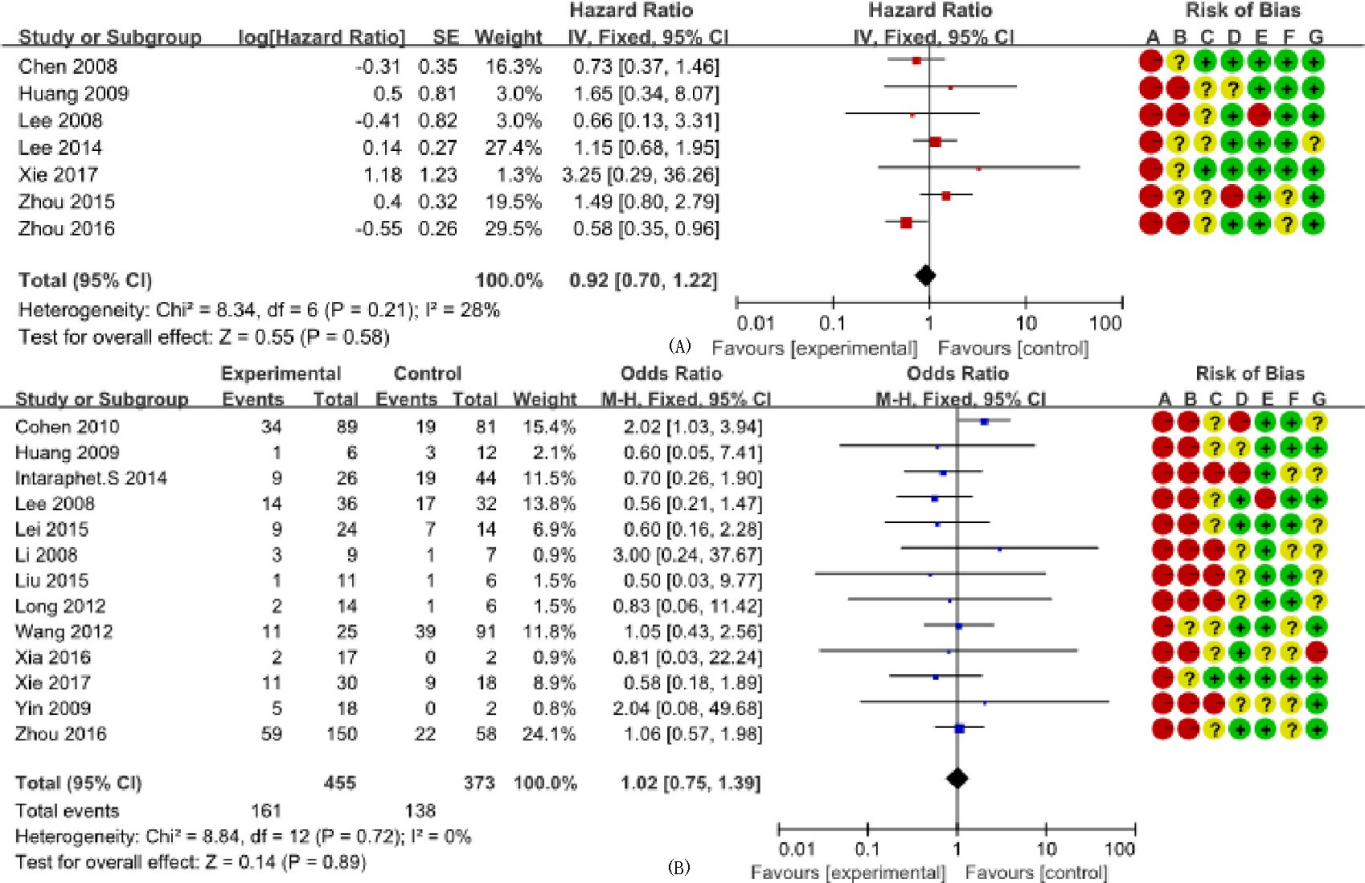
Forest plot of adjuvant radiotherapy (+/-) and overall survival and survival rate of small cell carcinoma of the cervix patients. (A) studies reporting hazard ratios; and (B) studies reporting odds ratios. SE: standard error; CI: confidence interval.

We determined the clinicopathological factors associated with the prognosis of SCCC through the interpretation of forest plots (Figs 2–12). The risk of mortality for stage IIb-IV SCCC was 2.63 times (95% CI: 2.13–3.24) that for stage Ia-IIa, that for diameter > 4 cm was 3.07 times (95% CI: 1.80–5.23) that for diameter ≤ 4 cm, and that for stromal infiltration > 2/3 was 1.99 times (95% CI: 1.33–2.97) that for stromal infiltration ≤ 1/3. Similarly, lymph node metastasis, parametrial invasion, and positive margins had hazard ratios of 1.69, 2.4, and 4.09, respectively. Neoadjuvant chemotherapy (NACT) and chemotherapy also reduced the risk of death.

On the other hand, the long-term survival rates represented by ORs also provided evidence that patients with stage Ia-IIa, no lymph node metastasis, and who received adjuvant chemotherapy had increased long-term survival, as represented by ORs of 3.72 (95% CI: 2.46–5.62), 2.09 (95% CI: 1.18–3.71), and 1.48 (95% CI: 1.02–2.16), respectively. However, in some studies, the number of cases (N) was low, and we used stratified analysis of FIGO staging and adjuvant radiotherapy (ART), and determined that, when N < 30, OR _FIGO_ = 2.35 (95% CI: 0.48–11.46) and OR _ART_ = 1.09 (95% CI: 0.36–3.26), and when N ≥ 30, OR _FIGO_ = 3.85 (95% CI: 2.51–5.90) and OR _ART_ = 1.02 (95% CI: 0.74–1.40). Although studies with N < 30 tended to have decreased or increased ORs, the OR of studies with N > 30 was close to the combined result, so the results were relatively stable.

**Fig 13:**
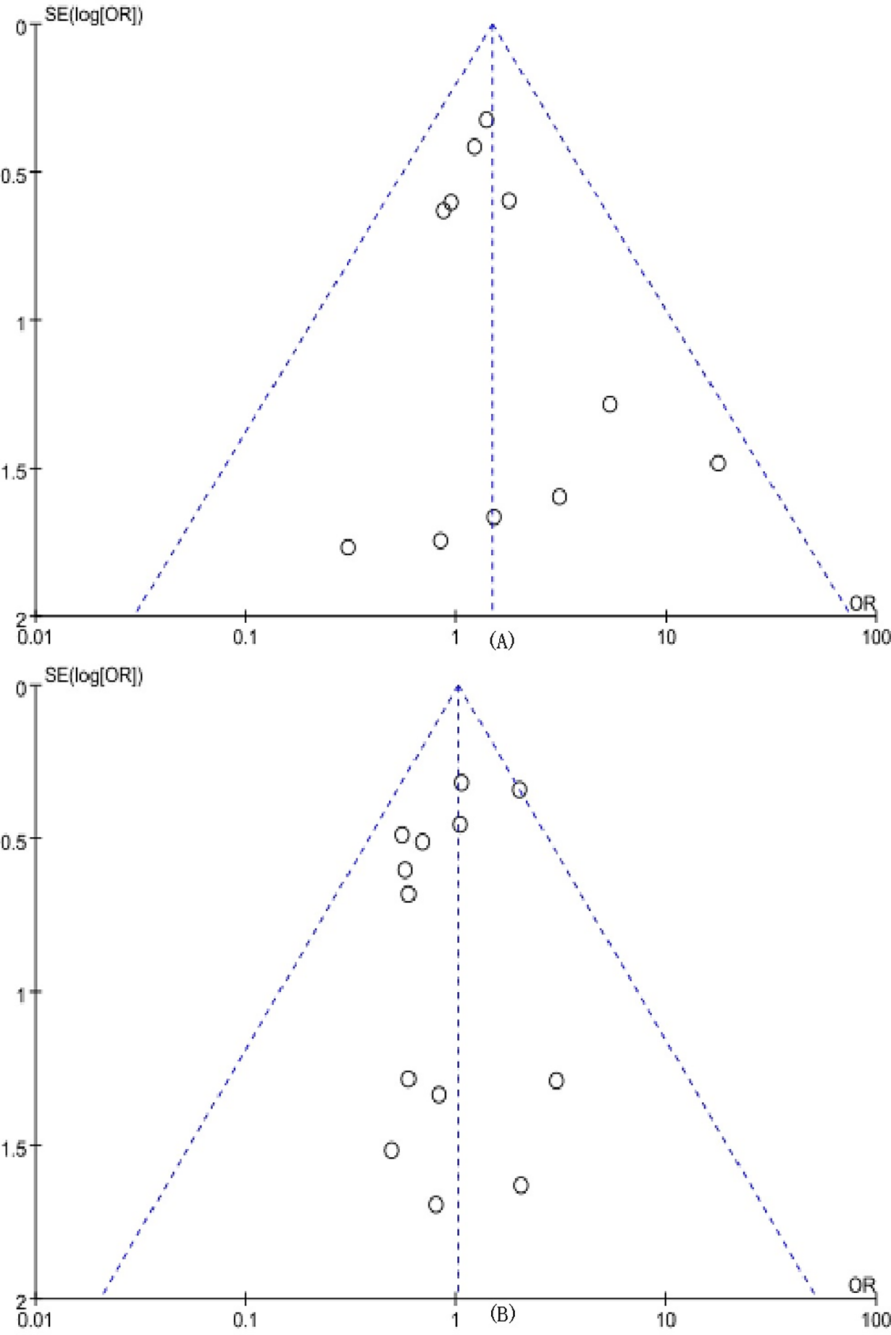
Publication bias. (A) Funnel plot of publication bias regarding the meta-analysis of adjuvant chemotherapy on survival rate. (B) Funnel plot of publication bias regarding the meta-analysis of adjuvant radiotherapy on survival rate.

Based on the survival rate of patients receiving adjuvant chemotherapy and ART, visual assessment revealed a roughly symmetrical distribution, indicating a low risk of publication bias in the meta-analysis (Fig 13). Similarly, the results of Egger’s, Begg’s, and Metatrim tests did not reveal any significant potential publication bias (the P-values of adjuvant chemotherapy and ART in the Egger’s, Begg’s, and Metatrim tests were 0.407, 0.640, and 0.577, and 0.219, 0.583, and 0.578, respectively).

## Discussion

We determined that, in patients with SCCC, FIGO staging, tumor diameter, lymph node metastasis, deep stromal infiltrates, parametrial infiltration, margins, NACT, and chemotherapy were important prognostic factors. However, this somewhat differed from previous studies. For example, other studies have shown that radiotherapy is a clinically important adjuvant therapy, but we did not find a significant correlation with prognosis in this study.

Histopathological biopsy is required for a definitive diagnosis of SCCC. In 2005, Tsunoda et al.[25] developed the following pathological diagnostic criteria of SCCC: tumor cells are of small round or spindle shape and lack cytoplasm; nuclei are deeply stained, chromatin appears as fine granules, and nucleoli are not obvious; cancer cells grow in a diffuse manner, or may form a nest, beam, or cord shape, with a fence shape or protrusion in the peripheral area; and often accompanied by necrosis, and mixed with squamous cell carcinoma or adenocarcinoma in some cases, such that the proportion of small cell carcinoma cannot be determined. Immunohistochemistry is an important auxiliary method for SCCC diagnosis. Commonly used immunological markers are similar to those used in other types of small cell carcinoma, including neuronal specific enolase (NSE), chromogranin A (CgA), synaptophysin (Syn), cytokeratin, nerve cell adhesion molecule (CD56), and epithelial cell markers, including carcinoembryonic antigen. However, at present, there is no single indicator with satisfactory sensitivity and specificity. In an immunohistochemical study conducted by Tsunoda et al., in 11 patients, the positive rates of NSE, Syn, and CD56 were 81.8%, 72.7%, and 54.5%, respectively, and the sensitivity of NSE was relatively good, although its specificity was poor. In another immunohistochemical study conducted by Li et al.[26], 25 patients with SCCC had positive rates of NSE, Syn, CD56, and CgA of 100.0%, 96.0%, 68.0%, and 76.0%, respectively, with higher positive rates of NSE, Syn, CD56, and CgA being considered more valuable for the diagnosis of SCCC and the differential diagnosis of cervical malignancies. Liao et al[17]. conducted a retrospective analysis and systematic review of 293 patients, and found that the 3-year survival rate was 46.8% in CgA-negative patients but only 30.0% in CgA-positive patients, with the mortality rate of CgA-positive patients being 1.81 times that of CgA-negative patients. Although the results were not significant, CgA was associated with a poorer prognosis for SCCC. Therefore, CgA positivity may be a prognostic factor of SCCC. However, owing to the lack of a universal indicator for immunohistochemical examinations of SCCC in many studies, as well as the incompleteness of the reported studies, an analysis could not be performed in the current study.

In this study, some clinicopathological characteristics, such as advanced FIGO stage, tumor diameter > 4 cm, presence of lymph node metastasis, deep stromal invasion, positive parametrial invasion, and positive resection margins appeared to be factors for poor SCCC prognosis. Many studies have shown that the 5-year survival rate is 31.6–36.4% for early-stage SCCC and 0–14% for advanced stage SCCC[27,28]. Our study indicated that tumor stage was an important factor of SCCC, and the risk of death increased by 2.63–3.72 times in patients with advanced stages compared with those with early stages. In patients with FIGO early stage disease, lymph node metastasis is considered an important prognostic factor, and the poor prognosis in some cases of SCCC may be related to early-stage lymph node metastasis[29]. In this study, heterogeneity was observed in the pooling of data of lymph node metastasis. A subgroup analysis of the study population showed that Taiwan was the source of this heterogeneity, which implies the existence of clinical and methodological heterogeneity. Survival analysis showed that the risk of death in patients with positive lymph node metastasis was increased 2.09 times. However, a study by Wang et al.[15] showed that if a patient receives surgical treatment, lymph node metastasis cannot be used as a prognostic factor. In addition, with regard to lymphovascular space invasion, clinical heterogeneity may exist, as determined in the study by Lee et al[18]. However, differences exist in the prognostic factors reported by previous retrospective analyses with regard to deep stromal invasion, lymphovascular space invasion, parametrial involvement, and resection margin status.

There has been controversy among researchers concerning the clinical treatment criteria of SCCC. This is mainly attributed to the rarity of this disease, which has resulted in inadequate prospective studies of different treatment models. At present, combination therapy involving surgery, chemotherapy, and radiotherapy is mainly utilized. The scope of surgery and surgical methods for SCCC are determined based on the clinical staging and scope of the tumor, which is consistent with methods used for other histological types of cervical cancers. The surgical method for Stage I-IIA SCCC is extensive radical hysterectomy plus pelvic and para-aortic lymphadenectomy[30]. As stage IIB-IV SCCC is prone to pelvic or distant local recurrence, clinicians have applied radiotherapy and chemotherapy, which are effective in controlling local lesions and reducing recurrence and metastasis. However, there is no uniform standard for radiotherapy and chemotherapy regimens for SCCC at present, and the chemotherapy regimens and treatment experience of small cell lung cancer have been used as a reference in most cases. In a controlled clinical study conducted by Chen et al.[31] in 110 patients who underwent initial surgery and 34 patients who underwent initial radiotherapy, it was found that, in most stage I-IIA patients, radiotherapy combined with at least 5 cycles of platinum-based chemotherapy seemed to be associated with a better survival rate than initial surgery. On the contrary, Lee et al. believed that initial chemotherapy combined with surgery or radiotherapy is conducive to improving prognosis in early-stage patients. In addition, Ruiz et al.[32] found that remission of stage I SCCC occurred with the use of 3 treatment models in a particular sequence (initial treatment with cisplatin and whole pelvic radiation, followed by brachytherapy for residual disease, and finally cisplatin and etoposide). These studies suggest that adjuvant chemotherapy is beneficial to the prognosis of SCCC, which is consistent with the results of this meta-analysis. Li et al.[33] found that patients who underwent NACT plus radical surgery had a lower rate of distant recurrence compared with those who underwent radical surgery alone (P = 0.029), and deduced that although platinum-based NACT cannot improve the survival rate of SCCC, it can control distant recurrence[34]. In a retrospective analysis of 13 patients with SCCC receiving a TPB regimen (topotecan, paclitaxel, and bevacizumab) and 21 patients who did not receive TPB conducted by Frumovitz et al.[35], it was found that the median progression-free survival was 7.8 months in the TPB group and 4.0 months in the non-TPB group (P = 0.001), suggesting that a TPB regimen improves survival. Although very few studies on NACT were included in this study, the results show that NACT is closely associated with prognosis.

The biggest limitation of this study is the lack of large-scale clinical experimental data based on exploration of prognostic risk factors and treatment models under different SCCC treatment modalities.

## Conclusions

Recently, the comprehensive treatment regimen for SCCC recommended by Gadducci et al. [36], which suggests that patients with stage IA–IIA1 SCCC should undergo surgery plus adjuvant chemotherapy (epirubicin + cisplatin; PE regimen), has been widely recognized. In addition, for patients with lymph node metastasis, parametrial invasion, and positive surgical margins, cisplatin-based concurrent chemoradiotherapy can be added, while for patients with stage IB2–IIA2 SCCC, radical surgery should be performed after 3 cycles of NACT (PE regimen). Patients with a complete response (lymph node-negative and complete remission of tumor) and those with an optimal partial response (persistent residual disease with stromal invasion < 3 mm and lymph node-negative) should receive 3 cycles of adjuvant chemotherapy (PE regimen). Patients with residual cervical disease with stromal invasion ≥ 3 mm and who are lymph node-positive should receive cisplatin-based concurrent chemoradiotherapy, followed by 3 cycles of adjuvant chemotherapy (PE regimen). Patients with Stage IIB-IVA disease should receive NACT for 3 cycles (PE regimen), followed by cisplatin-based concurrent chemoradiotherapy (pelvic and aortic areas) and an additional 3 cycles of chemotherapy. For patients with stage IVB SCCC, chemotherapy should be considered for the initial treatment, followed by palliative radiotherapy in the pelvic area. In patients with highly selective lung metastasis, prophylactic cranial irradiation can be considered.

## Supporting information

S1 Checklist PRISMA checklist. A PRISMA checklist for this systematic review.
(DOC)

S1 Flow Diagram PRISMA 2009 flow diagram. A PRISMA 2009 flow diagram for this systematic review.
(DOC)

S1 Text Nosgen. The Newcastle-Ottawa Scale (NOS) as a quality assessment method in this systematic review.
(PDF)
