# Supplementary material for: Influence of clinicopathological characteristics and comprehensive treatment models on the prognosis of small cell carcinoma of the cervix: A systematic review and meta-analysis
Source: PLoS One. 2018 Apr 11;13(4):e0192784. doi: 10.1371/journal.pone.0192784 (PMC5894955; doi:10.1371/journal.pone.0192784)
Supplement: S1 Flow Diagram — A PRISMA 2009 flow diagram for this systematic review. (DOC) [file pone.0192784.s002.doc]

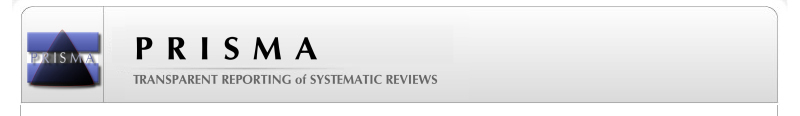
**PRISMA 2009 Flow Diagram**

**Screening**

**Included**

**Eligibility**

**Identification**

Records identified through database searching
(n = 1714 )

Additional records identified through other sources
(n = 1 )

Records after duplicates removed
(n = 373)

Records screened
(n = 1342 )

Records excluded
(n = 1155 )

Full-text articles assessed for eligibility
(n = 187 )

Full-text articles excluded, with reasons
(n = 167 )

Studies included in qualitative synthesis
(n = 20 )

Studies included in quantitative synthesis (meta-analysis)
(n = 20 )
